# MRI Findings in a Rare Case of Left-Sided Obstructed Hemivagina and Ipsilateral Renal Anomaly (OHVIRA) Syndrome in a 21-Year-Old Female

**DOI:** 10.7759/cureus.46704

**Published:** 2023-10-09

**Authors:** Iram Saifi, Shivali V Kashikar, Pratap Parihar, Azeem I Saifi, Khizer K Ansari

**Affiliations:** 1 Radiology, Jawaharlal Nehru Medical College, Datta Meghe Institute of Higher Education and Research, Wardha, IND; 2 Radiation Medicine, Jawaharlal Nehru Medical College, Datta Meghe Institute of Higher Education and Research, Wardha, IND; 3 Medicine and Surgery, Jawaharlal Nehru Medical College, Datta Meghe Institute of Higher Education and Research, Wardha, IND

**Keywords:** uterine didelphys with ipsilateral renal agenesis, mri findings in ohvira, complex mullerian anomaly, hww syndrome, ohvira syndrome

## Abstract

Obstructed hemivagina and ipsilateral renal anomaly (OHVIRA) is a complex Mullerian and Wolffian duct anomaly, which is difficult to diagnose before puberty. We present a rare case of a congenital syndrome known as OHVIRA in a 21-year-old female who came with complaints of intermittent type of lower abdominal pain, dysmenorrhea, and oligomenorrhea with frequent visits to different hospitals without any radiological investigations done. Early magnetic resonance imaging (MRI) investigations helped her in diagnosing and managing this syndrome.

## Introduction

Obstructed hemivagina and ipsilateral renal anomaly (OHVIRA) syndrome, also known as Herlyn-Werner-Wunderlich (HWW) syndrome, is a rare congenital anomaly including a triad of obstructed hemivagina, ipsilateral renal anomaly, and Mullerian duct anomaly such as uterine didelphys. The prevalence of this syndrome is approximately 0.1%-3%. The renal anomaly present in this syndrome is usually renal agenesis. However, sometimes, renal duplication and multicystic dysplastic kidney can also be associated in place of renal agenesis. Patients usually present during puberty or in early menarche. Patients usually visit hospitals related to issues such as lower abdominal pain and menstrual irregularity [1]. The first investigation usually done is ultrasonography, which helps in giving clues about this condition, but magnetic resonance imaging (MRI) helps in identifying this rare syndrome accurately and also guides in prompt management so that early treatment helps in preventing complications such as pyosalpinx, infertility, adhesions, and endometriosis due to retrograde menstruation in such a condition. Early diagnosis and treatment help preserve the fertility of the patient, which can also improve the social and psychological burden of the person, as well as the family. The counterpart of this syndrome in males is obstructed seminal vesicle and ipsilateral renal agenesis (OSVIRA) in which, along with ipsilateral renal anomaly, there is the presence of a unilateral seminal vesicle cyst and obstructed ejaculatory duct.

## Case presentation

A 21-year-old female came to the hospital with complaints of lower abdominal pain on and off, recurrent, and recently for the last two months. The patient has had similar episodes of dull aching pain in the past for almost a year. The patient had dysmenorrhea and hypomenorrhea in each menstrual cycle. The patient has no complaints of abdominal trauma and no bowel and bladder complaints. Appetite is normal. The patient has no history of any addiction. Her local examination was within normal limits. Respiratory, musculoskeletal, central nervous, cardiovascular, and gastrointestinal system examinations were within normal limits. The patient was advised for non-contrast magnetic resonance imaging of the abdomen and pelvis for recurrent abdominal pain for 2-3 months and menstrual complaints. She was nil per oral for four hours. MRI was performed on a Philips 3 T MRI machine (Amsterdam, Netherlands). The patient was positioned in a lying down supine position with the head toward the magnet, and the body coil was placed from the nipple to a few inches below the pubic symphysis. The sequences taken were axial T2 fat-suppressed (FS) full field of view (FOV), axial T1 full FOV, coronal T2 full FOV to include the kidneys, gradient echo imaging (GRE), coronal Dixon, axial spectral adiabatic inversion recovery (SPAIR) sequence, and small FOV in relation to the uterus: axial T1, T2-weighted image (T2WI), sagittal T1, T2WI, coronal T1, and T2WI. The complete MRI scan took almost 60 minutes.

**Figure 1:**
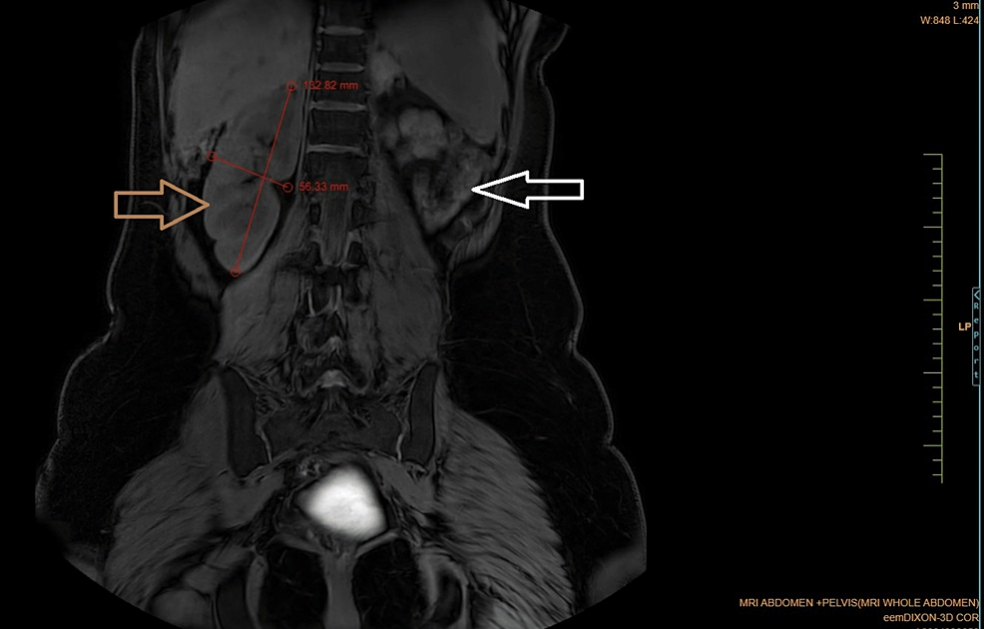
Coronal Dixon imaging showing the absence of the left kidney (white arrow) and right kidney (brown arrow) measuring 132.82 × 56.33 mm suggestive of compensatory hypertrophy. Note: Authors' original image

**Figure 2:**
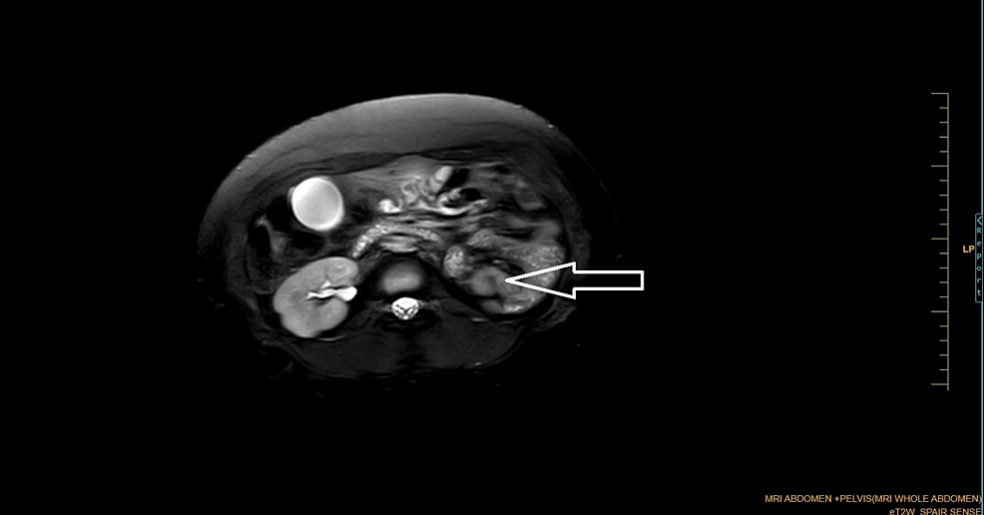
Axial T2W SPAIR imaging showing the absence of the left kidney. Note: Authors' original images T2W, T2-weighted; SPAIR, spectral adiabatic inversion recovery

**Figure 3:**
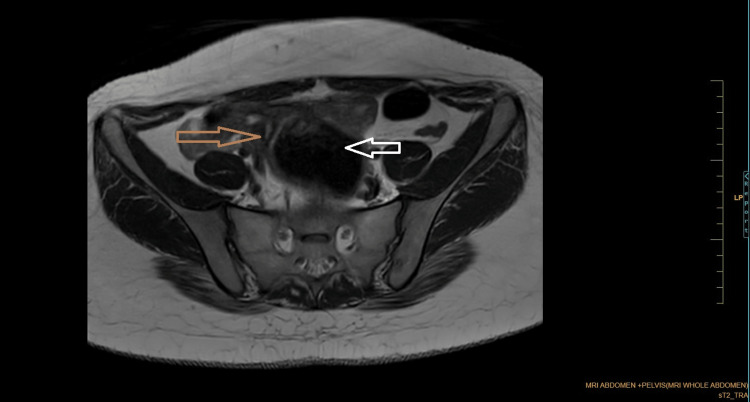
Axial T2WI showing two widely separated divergent uterine horns with fundal cleft. There is evidence of cystic collection appearing hypointense on T2 communicating with the left-sided cervix and uterus (white arrow). On the right side, normal uterus and cervix are present but compressed and pushed laterally by hematocolpos (brown arrow). Note: Authors' original image T2WI: T2-weighted image

**Figure 4:**
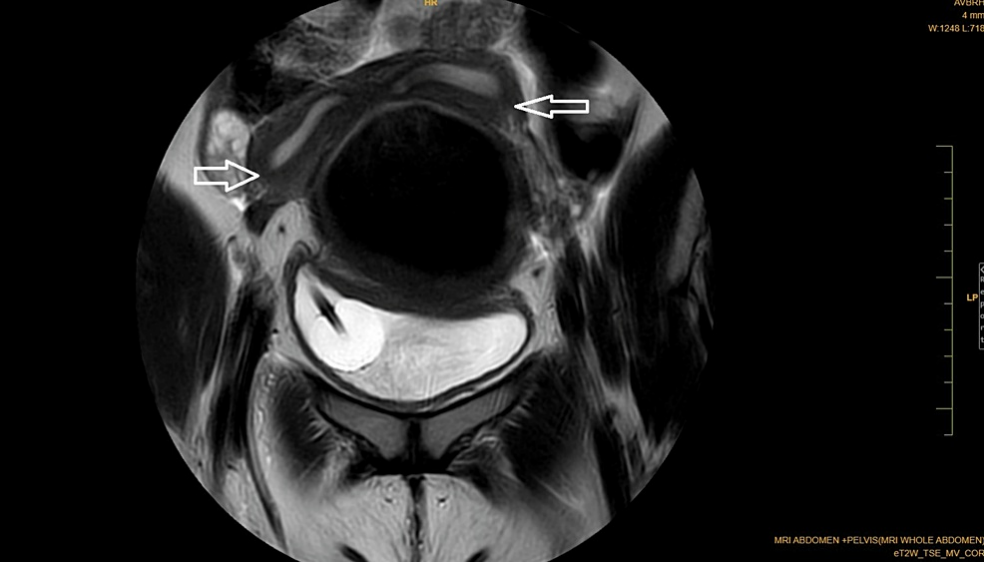
Coronal T2WI showing two separated divergent uterine horns (white arrows) with fundal cleft suggestive of uterine didelphys with hematocolpos. The urinary bladder shows a Foley bulb in situ. Note: Authors' original image T2WI: T2-weighted image

**Figure 5:**
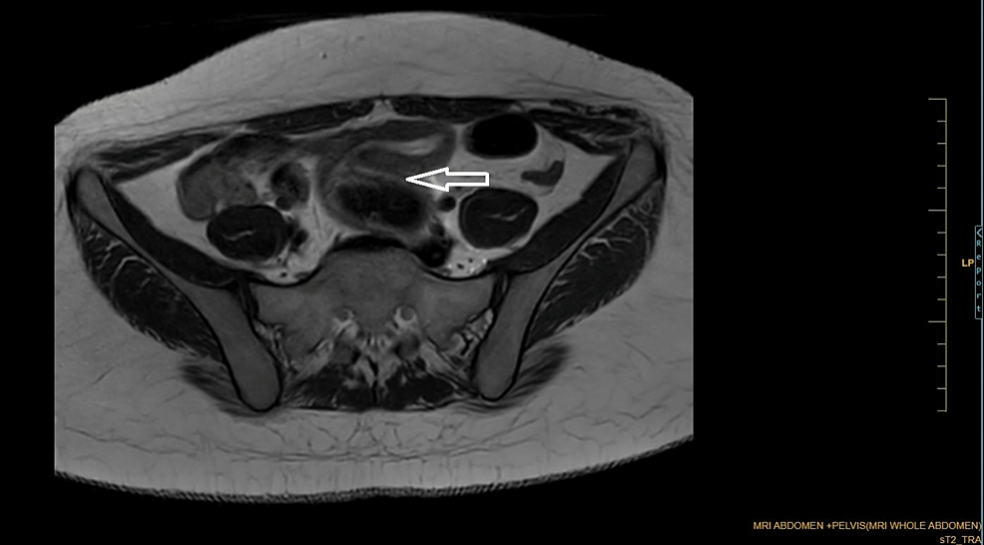
Axial T2WI showing dilated vagina with hypointense collection communicating with the left-sided uterus suggestive of hematocolpos. Note: Authors' original image T2WI: T2-weighted image

**Figure 6:**
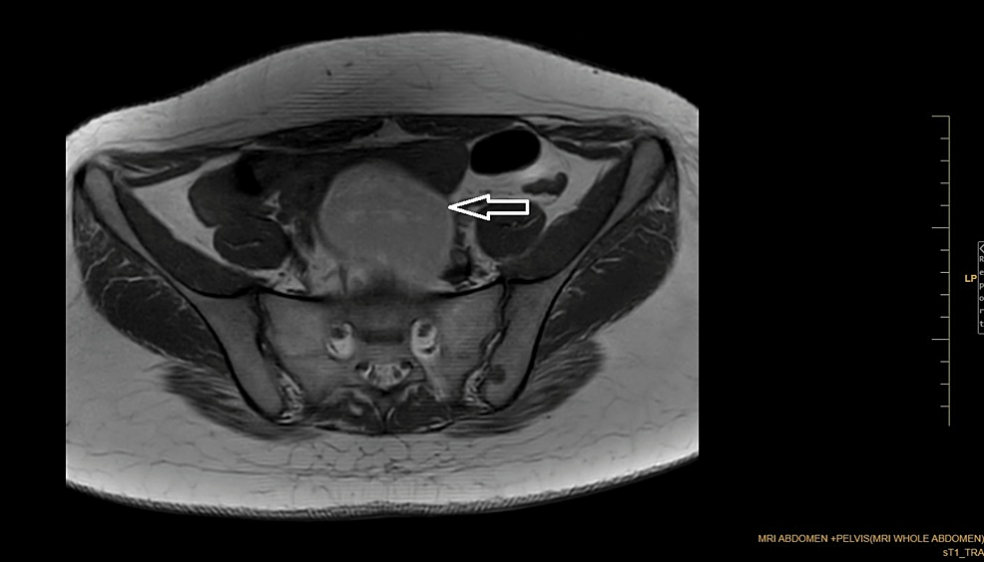
Axial T1WI showing hyperintense collection in the vagina suggestive of hematocolpos. Note: Authors' original image T1W1: T1-weighted image

**Figure 7:**
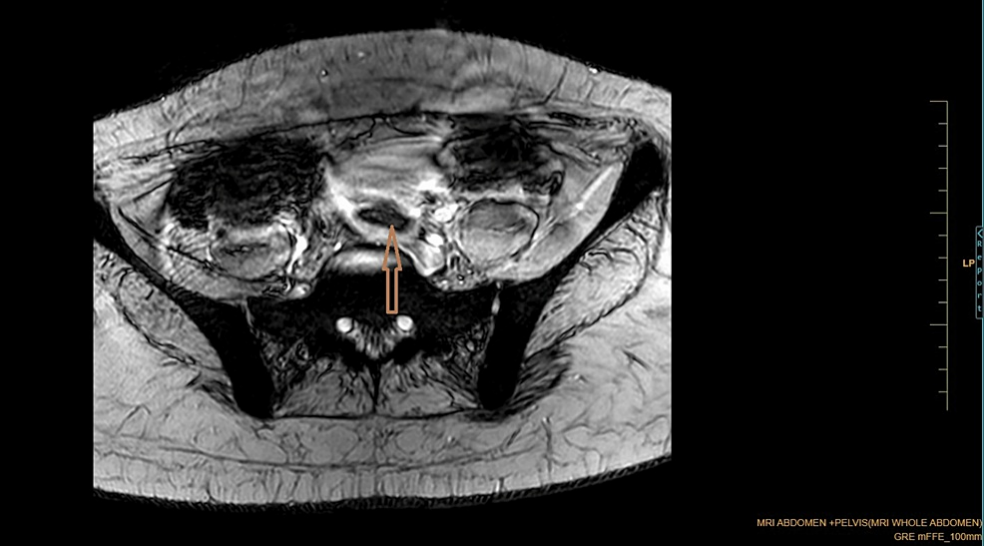
Axial GRE imaging showing blooming in the collection inside the vagina suggestive of hematocolpos. Note: Authors' original image GRE: gradient echo imaging

**Figure 8:**
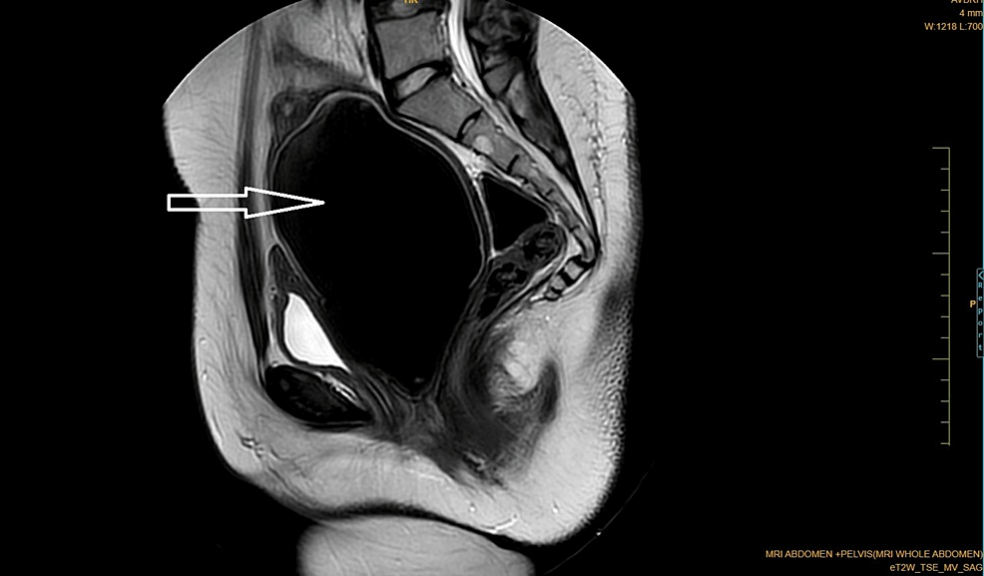
Sagittal T2WI showing large well-defined hypointense cystic collection inside the vagina. Note: Authors' original image T2WI: T2-weighted image

Coronal Dixon MRI and axial T2WI show the absence of the kidney on the left side. The right kidney measures 132.82 × 56.33 mm suggestive of (s/o) compensatory hypertrophy (Figures 1, 2). There is no presence of any ectopic kidney in the pelvis or any other site in the abdomen. In axial and coronal T2WI, there is the presence of two widely separated divergent uterine horns with fundal cleft suggestive of uterine didelphys (Figures 3, 4). On the left side, there is the presence of a large cystic collection appearing hypointense on T2, hyperintense on T1, and blooming on GRE s/o hematocolpos (Figures 3, 5-8). On the right side, normal uterus and cervix are present but compressed and pushed because of hematocolpos (Figure 3). So the findings in our case include an absent left kidney with didelphys uterus and obstructed hemivagina, which make it a rare case of Herlyn-Werner-Wunderlich syndrome, also known as OHVIRA. In our case, the patient went to the gynecology department and was counseled about this syndrome, fertility problems, and related complications. Emergency vaginal septum resection was performed.

## Discussion

OHVIRA is a rare congenital anomaly of the genitourinary system. It was first mentioned in history in the 1970s by three scientists named Herlyn, Werner, and Wunderlich. That is why it is also known as HWW syndrome. This syndrome consists of a triad of obstructed hemivagina, ipsilateral renal anomaly, and uterine didelphys; that is why it is called OHVIRA [2-4]. This syndrome is basically related to Mullerian duct anomalies, so the understanding of embryology is very important in understanding and diagnosing this condition. There are two theories related to vaginal development, the classic theory and Acién theory. The classic theory demonstrates that the vagina is divided into upper and lower parts, in which the upper part of the vagina is developed by Mullerian ducts and the lower part is developed by sinovaginal bulbs of the urogenital sinus. This theory can tell us about Mullerian duct anomalies, but it cannot explain the development of anomalies such as OHVIRA and the association of Mullerian duct anomalies with kidney pathology [5]. Another scientist named Acién gave a different theory. He explained that there are two ducts called; one is Mullerian ducts, also known as paramesonephric ducts. Another duct is the Wolffian duct, also known as the mesonephric duct. The uterus and cervix are formed from joined Mullerian ducts, and the vagina is formed from the Wolffian duct. But the vaginal lining consists of paramesonephric cells, which are derived from the paramesonephric tubercle, but the entire vagina is not developed from paramesonephric ducts. And since vaginal and renal development occurs from the Wolffian duct, if there is any error in derivation from one side of the Wolffian duct, it will affect the same side of the kidney and vagina or both. This theory is given by Acién and helps in understanding complex anomalies such as OHVIRA [6,7]. Near the fifth week of gestation, the Wolffian duct gives rise to the metanephric diverticulum. If this does not form, it will lead to the agenesis of the same side of the kidney and hemivagina. Mullerian development is lateral to the Wolffian duct at around nine weeks. After crossing the Wolfman duct, they come in midline and fuse to form the cervix and uterus. If they do not fuse, it results in uterine didelphys. So anomalies in the Wolffian duct and the fusion of two Mullerian ducts will give rise to OHVIRA.

Normally, the incidence of a right-sided anomaly in OHVIRA is higher compared to a left-sided anomaly. Left-sided anomalies are rare. A detailed history, examination, and radiological investigation can give rise to clues about this condition. In diagnosing OHVIRA syndrome, ultrasonography and MRI are considered important. MRI is even superior to ultrasonography to confirm its findings and give a detailed extent of anomalies [8,9]. Some gynecologists also prefer laparoscopy when MRI results are not well understood.

Treatment is usually surgical including the resection of vaginal septum to relieve hematocolpos [10]. In our case, the patient was thoroughly explained about the procedure in her language, and possible damage to the hymen was also addressed to the patient and family since she was a virgin and unmarried. A well-written informed consent was taken. A vaginoscopy was done to visualize the longitudinal septum. The procedure was done in the operation theatre through a vaginal route. The vagina was dilated by vaginal dilators to get a better view of the resection of tissue. The longitudinal distal part of the septum was cut and resected followed by a drainage of hematocolpos, and a single vaginal canal was created.

It is very important to diagnose this condition as early as possible because it can lead to various complications such as abscess in the vagina, cervix, and uterus. If the condition is not treated at the appropriate time, it will lead to infection by different organisms, eventually leading to vaginal abscess formation. There are more chances of developing endometriosis due to the risk of retrograde menstruation. Pyosalpinx, adhesions, and infertility are other complications. In such patients, there is a concern regarding fertility. Infertility is the most important concern for parents since it is associated with psychological and social issues. If proper care is not taken early, pyosalpinx and adhesions can cause infertility. Since the patient has a didelphic uterus, the patient can become pregnant and can deliver normally. However, the chances of premature labor and abortions are higher in such cases compared to the general population [11]. There is a risk of developing vesicoureteric reflux to the contralateral kidney because of the absence of another kidney. Renal anomalies such as dysplastic kidneys also pose a risk of early development of chronic kidney disease. In such a case, a follow-up ultrasound is also necessary to assess the risk from time to time.

## Conclusions

OHVIRA is a rare Mullerian duct anomaly, especially left-sided, which is even rarer. In our case, the patient presented with complaints regarding her menstrual cycle, lower abdominal pain, and frequent visits to different doctors and hospitals without having any radiological investigations done until the age of 21. The first visit to the radiology department and a detailed MRI investigation not only helped her in diagnosing the syndrome but also helped her to plan for immediate treatment and her future fertility-related issues. This case helped us learn the lesson that pelvic pain in postmenstrual females should raise suspicion for some pelvic pathology and should not be ignored by giving symptomatic treatment only. This case also highlights the importance of renal anomalies in association with genital anomalies since the developmental lineage is the same. This recommends the clinician to take a thorough history of the patient's abdominal pain, its duration, and radiological investigations done in the past. It highlights the importance of gynecological examination in the case of lower abdominal pain in females, and a delay should not be made for at least one sonographic assessment. This case also demonstrates the need for magnetic resonance imaging in identifying pathologies accurately related to the pelvis and going for immediate intervention.
